# Preparation of PLA/chitosan nanoscaffolds containing cod liver oil and experimental diabetic wound healing in male rats study

**DOI:** 10.1186/s12951-020-00737-9

**Published:** 2020-11-30

**Authors:** Payam Khazaeli, Maryam Alaei, Mohammad Khaksarihadad, Mehdi Ranjbar

**Affiliations:** 1 Pharmaceutics Research Center, Institute of Neuropharmacology, Kerman University of Medical Sciences, P.O. Box: 76175-493, Kerman, 76169-11319 Iran; 2 Faculty of Pharmacy, Kerman University of Medical Sciences, Kerman, Iran; 3 Student Research Committee, Kerman University of Medical Sciences, Kerman, Iran; 4 Neuroscience Research, and Physiology Research Centers, Kerman University of Medical Sciences, Kerman, Iran

**Keywords:** Poly lactic acid/chitosan nanoscaffolds, Cod liver oil, Electrospinning, Diabetic foot ulcer

## Abstract

Diabetes mellitus is one of the most common metabolic disorders. One of the important metabolic complications in diabetes is diabetic foot ulcer syndrome, which causes delayed and abnormal healing of the wound. The formulation of nanoscaffolds containing cod liver oil by altering the hemodynamic balance toward the vasodilators state, increasing wound blood supply, and altering plasma membrane properties, namely altering the membrane phospholipids composition, can be effective in wound healing. In this study, electrospinning method was used to produce poly lactic acid/chitosan nanoscaffolds as a suitable bio-substitute. After preparing the nanoscaffolds, the products were characterized with dynamic light scattering (DLS), transmission electron microscopy (TEM) and scanning electron microscopy (SEM). Also optical properties of polymer and comparison between adsorption between single polymer and polymer-drug calculated with UV−Vis spectra. The structure and functional groups of the final products were characterized by Fourier-transform infrared spectroscopy (FT-IR) and energy dispersive spectroscopy (EDAX) as elemental analysis. The results showed that the optimum formulation of cod liver oil was 30%, which formed a very thin fiber that rapidly absorbed to the wound and produced significant healing effects. According to the results, poly lactic acid/chitosan nanoscaffolds containing cod liver oil can be a suitable bio-product to be used in treating the diabetic foot ulcer syndrome.
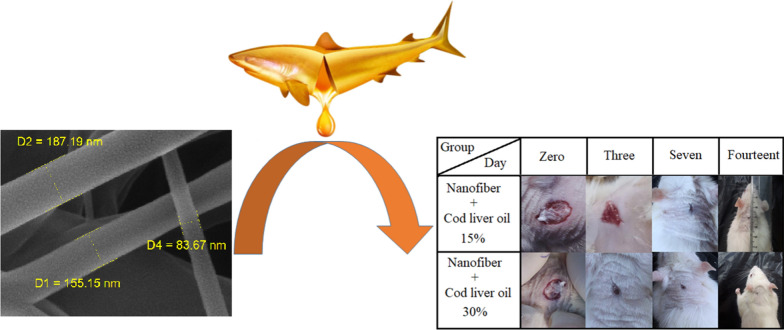

## Research highlights

- Poly lactic acid/chitosan nanoscaffolds were synthesized using microwave-assisted electrospinning process.
- Nanoscaffolds showed high potential in wound healing recovery after 14 days.
- PLA/chitosan nanoscaffolds containing 30% cod liver oil were synthesized with the size of about 50–150 nm.
- Wound area indicated that there was significant improvement in wound surface on the 14th day.

## Introduction

The global prevalence of diabetes has increased dramatically over the past 2 decades [1]. Diabetes mellitus is the most common heterogeneous metabolic disorder [2], which is associated with a disorder in the metabolism of sugars, lipids, and proteins and is characterized by elevated blood glucose or insulin response to tissues [3, 4]. Patients suffering from diabetes mellitus have limited ability to stimulate the immune response and are very susceptible to infection and at the risk of terminal limb amputation and recurrence of the wound [5]. Fatty acids have physiological and pathological roles in diseases such as atherosclerosis [6, 7], inflammation [8], or normal wound healing [9, 10], The effect of fatty acids on wound healing is through alterations in plasma membrane properties [11, 12], such as changes in membrane phospholipids composition [13–15], increased growth factor activity [16, 17], cell differentiation [18, 19], decreased eicosanoids production [20], and lipid mediators of inflammation [21], followed by reducing inflammation and producing interleukin-1 and collagen [22]. The cod liver oil as a rich source of omega-3 fatty acids has many potential effects on modulating various diseases, especially diabetes mellitus [23, 24], improvements in vasodilator property [25–27]. In many studies immune and allergic responses of rats was investigated for wound healing [28–30] Many scientific works have shown which cod liver oil accelerates many of the potential mechanisms involved in wound healing [31–33]. In recent years, new drug delivery systems such as nanofibers [34], nanoparticles [35], cell therapy, and stem cell [36] being used as alternative therapies for common pharmaceutical methods, which could reduce the need for continuous follow-up of the disease and increase the quality of treatment, have received great attention [37]. Nanotechnology has solved many concerns in the field of medicine due to dealing with materials that have unique properties on their surface [38–43]. Chitosan structures have a good crosslink structure for encapsulating drugs [44] and polylactic acid possesses properties such as the ability to form hydrogels in physiological conditions [45], mild gel degradation for a wound to heal successfully, and the growth and movement of nutrients [46]. In recent years, the science of nanotechnology has attracted particular attention from researchers in various fields of medicine and pharmaceuticals [47]. Nanofibers and nanoparticles can release the drug in a controlled approach for a long time [48]. These structures can act as an appropriate topical drug delivery system that can provide the appropriate drug concentration and other advantages of this system include the ability to transport hydrophilic and lipophilic drugs simultaneously depending on their structure [49]. Examples of natural polymers used in the fabrication of nanofibers with electrospinning method [50–53] include creatine [54], gelatin [55], cellulose [56], and polysaccharides such as chitosan and alginate. The synthesis of the PLA/ Chitosan nanofibers has been reviewed in recent studies [57–59]. Microwave irradiation as a cost-effective, eco-friendly, and high efficiency method is used for preparing nanoparticles for various applications [60], electrospinning process with high-voltage power, generate polymer fibers in nanometer dimensions which show unique physical and chemical properties [61]. In this study, new developments in the fabrication of nanoscaffold materials such as the microwave-assisted electrospinning process were applied to prepare and formulate poly lactic acid/chitosan containing cod liver oil as a suitable cost-effective method. Summary of the research on nanoscaffolds applications in wound healing recovery is displayed in Table 1. The results indicate that the synergistic effect quantity of the poly lactic acid/chitosan containing hydrogel is the key factor in obtaining suitable biological wound for wound dressing.

**Table 1:** Summary of researches about nanoscaffolds applications in wound healing recovery

Types of nanofibers	Particle size (nm)	Synthesis method	Characterization	References
PVA–clay nanocomposite	60–100	Cyclic freezing–thawing	SEM, XRD, tensile modulus	[63]
Chitosan	20–40	Coprecipitation	FTIR, TGA, DSC, SEM and TEM	[64]
Cerium oxide nanoparticle-containing poly (ε-caprolactone)	50	Electrospinning	SEM, DLS, MTT	[65]
Halloysite and chitosan oligosaccharide	200	Composition	SEM, EDX, UHRTEM,, Zeta potential In vivo	[66]
Montmorillonite/chitosan	–	Solid state	HRTEM, XRD, XEDS, TGA	[67]

## Experimental

All materials and precursors used in this research work were pure without any impurities and were purchased directly from reputable commercial centers. Chitosan (CAS: 9012-76-4, MW Mol wt‎: ‎50,000 daltons based on viscosity, 99.90%), Polylactic acid (C(CH_3_) HC(=O) O–) and Dimethylformamide (DMF, MW; 73.095 g mol^− 1^) were purchased from Sigma Aldrich agents in IRAN). Polysorbate 80 (tween 80, C_64_H_124_O_26_, MW: 1.310 g/mol) was purchased from FLOKA company in Switzerland. NaOH (d: 2.13 g/ml, MW: 39.9971 g/mol, 99.99%) was purchased from Dr. Abidi company in IRAN. We purchased cod oil lever from institute of pharmaceutical services Razavi company. Xylazine and Ketamine for anesthesia and intraperitoneal tolerance in rats were purchased from Alfasan group of companies in Netherlands. Male rats were obtained from Kerman university of medical animal’s farm. Also this study received ethical approval from the local ethical committee of the kerman university of medical sciences as a thesis research at the faculty of pharmacy kerman university of medical sciences with number 1124. Male rat weighing 150–200 g was fed with standard diet and kept under 12:12 h light/dark cycles, at 20 ℃ and relative humidity of 25–30%. XRD patterns for crystalline phase detection were recorded by a Rigaku D-max C III, X-ray diffractometer using Ni-filtered Cu Ka radiation. Microscopic morphology and investigation of surface propertieso of the products were characterized by SEM (LEO 1455VP). The energy dispersive spectrometry (EDS) supplier analysis to determine the elements in the samples was studied by XL30. Transmission electron microscopy (TEM) images were obtained with a Philips EM208 transmission electron microscope with an accelerating voltage of 200 kV. Fourier transform infrared (FT-IR) spectra were recorded on Shimadzu Varian 4300 spectrophotometer in KBr pellets. To absorption evaluate samples ultraviolet–visible spectroscopy analysis was carried out using Shimadzu UV-2600 UV–Vis spectrophotometer.

### Preparing PLA/chitosan nanofibers

To prepare the polymer phase, at first, 0.2 g of PLA was dissolved in 18:3 ml ratio of deionized water and ethanol after heating and stirring at 50 °C and RPM 400 for 45 min. Then, 5 ml of NaOH (2 mol/l) was added to the above solution and this solution was heated at 60 °C and stirred for 30 min. In the next step, 0.05 g of chitosan was dissolved in 2:1 ml ratio of deionized water and dimethylformamide after 110 min. Subsequently, the solutions were transferred to a beaker and exposed to the microwave irradiation oven under the power of 450 W for 5 min. Regular cycles of the microwave irradiation were set to 30 s off and 60 s on. Finally, the solutions were placed in an environment free of contamination for 24 h to complete the crystallization process.

### Cod liver oil loading

First, 2 ml of polymeric solution was added to 100 µl of the drug with the concentrations of 15% and 30% by weight in the presence of 350 µl Tween as the surfactant agent and placed on the reflux system for 30 min at 50 °C and 500 rpm on the shaker for 15 min. PLA/chitosan nanoscaffolds containing cod liver oil were formed by an electrospinning device. Nanoscaffolds containing 30% w/w and 15% w/w cod liver oil were prepared at the speed of 2 ml/h; 12.1 V and jet rotation speed of 100 rpm were used to form the nanofibers.

### In vivo study

For the in vivo study, the male rats were divided into four groups (each group containing 6 mice weighing approximately 200 g). Animals were diabetic by the intraperitoneal injection of 60 mg/kg and their diabetes was confirmed after 3 days by measuring glucose using a glucometer. Then, after anesthetizing the rats with ketamine/xylazine, an ulcer about 1.5 cm in the area between the two scapula was created by punch biopsy and, then, the drug was positioned topically on each group. For this study, four groups of mice were divided into the following groups: Mice in Group 1 treated with nanofiber alone; Mice in Group 2 treated with cod oil only; Mice in Group 3 treated with nanofiber delivery system containing cod liver oil for wound healing; and Mice in Group 4 received no treatment (negative control). To induce diabetes, we injected strepotozocin 60 mg/kg intraperitoneally in rats. All the experimental procedures were carried out according to the protocols set for working with animals at Kerman University of Medical Science (Kerman, Iran).

## Result and discussion

### Physicochemical characteristics

**Fig. 1:**
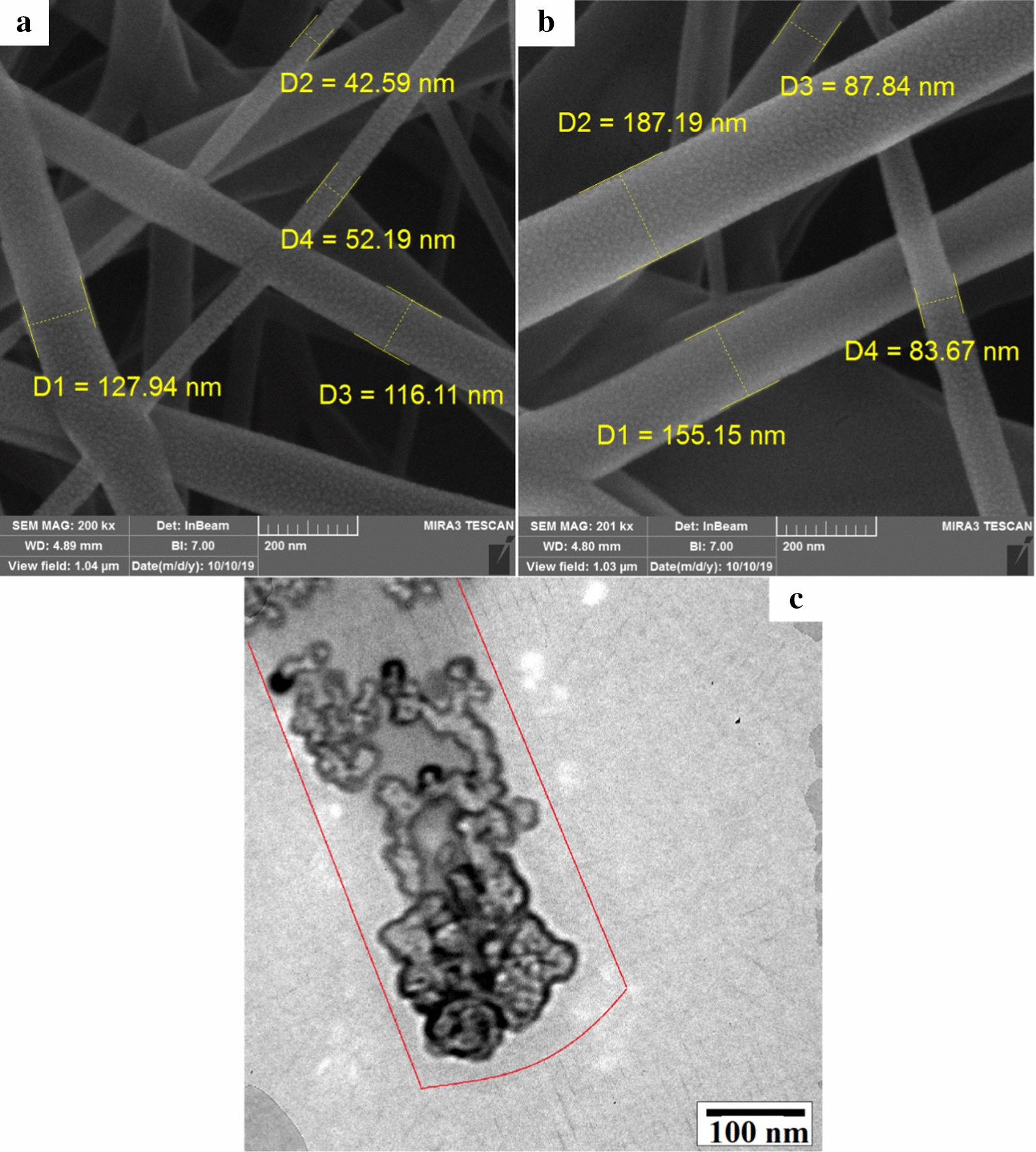
SEM images of the poly lactic acid/chitosan nanofibers containing cod liver oil 30% w/w cod liver oil (**a**), 15% w/w cod liver oil (**b**) and TEM image of the poly lactic acid/chitosan nanofibers containing 30% w/w cod liver oil

Morphological properties and surface features of the nanofibers were observed through scanning electron microscopy (SEM) images. SEM images with the approximate scaffold size of poly lactic acid/chitosan nanofibers containing 30% and 15% cod liver oil are displayed in Fig. 1a and b, respectively. As can be seen from the SEM images of the nanoscaffold structures, they were uniform and had no cracking along the formation. The nanoscaffold structures encapsulated different concentrations of cod liver oil without any systemic defects and the nanofibers containing 30% cod liver oil showed less diameter than 15% cod liver oil; this may be due to higher solubility of the polymer phase at higher concentrations of oil phase. According to SEM images, the average size of the diameter scaffold tube was estimated between about 50 and 150 nm. To investigate the three-dimensional (3D) images of the fibers, we did transmission electron microscopy (TEM). According to the TEM images, the cod liver oil was trapped uniformly in the spaces between PLA/chitosan nanoscaffolds. The oil phase ranges in the polymer phase were well-defined. The TEM image of 30% w/w cod liver oil cod liver oil distributed in poly lactic acid/chitosan nanoscaffolds is shown in Fig. 1c.

**Fig. 2:**
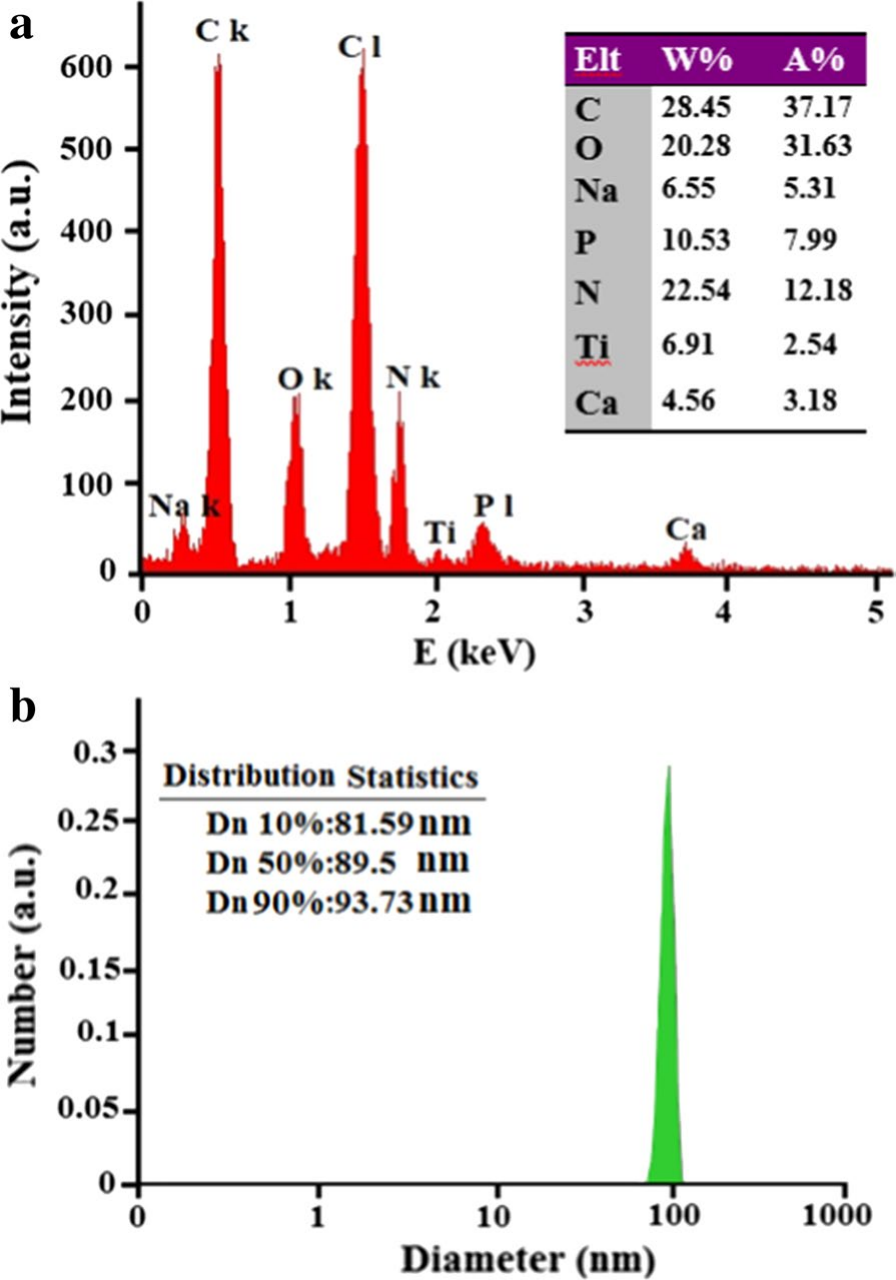
EDAX supplier analysis (**a**) and DLS data diagram after 15 min ultrasonic irradiation at 60 W (**b**) of the poly lactic acid/chitosan nanofibers 30% w/w containing cod liver oil

Energy dispersive spectroscopy (EDAX) is a suitable supplier analysis used for the semi-quantitative analysis of elements. This method is mainly used to obtain point chemical composition and quantitatively investigate the poly lactic acid/chitosan nanoscaffolds containing cod liver oil. About 37.17 percent of the total atomic weight of the final products was related to C atoms; this could be related to carbon atoms in poly lactic acid, chitosan, omega3 in cod liver oil, and Tween structures. The existence of the O, Na, and N atoms to the amount of about 31.63, 5.31, and 15.36 percent could be related to the existence of these atoms in poly lactic acid, chitosan, cod liver oil, Tween, NaOH, and dimethylformamide structures. The small amounts of Ti and Ca atoms could be related to the unpredictable impurities in the final products. The EDAX as a supplier analysis of the poly lactic acid/chitosan nanoscaffolds containing 30% cod liver oil is shown in Fig. 2a. The size distribution obtained from the nanoscaffolds was a plot of the relative intensity of light scattered by nanoscaffolds in various size classes and introduced as the intensity size distribution. Results related to the size distribution of the nanoscaffolds obtained from dynamic light scattering analysis showed good match with SEM images and estimated the size of the poly lactic acid/chitosan nanoscaffolds containing 30% cod liver after 15 min ultrasonic irradiation at 60 W fibers of about 50–150 nm in Fig. 2b. Also the size distribution of PLA/chitosan nanofibers without cod oil liver was calculated about 120 nm.

**Fig. 3:**
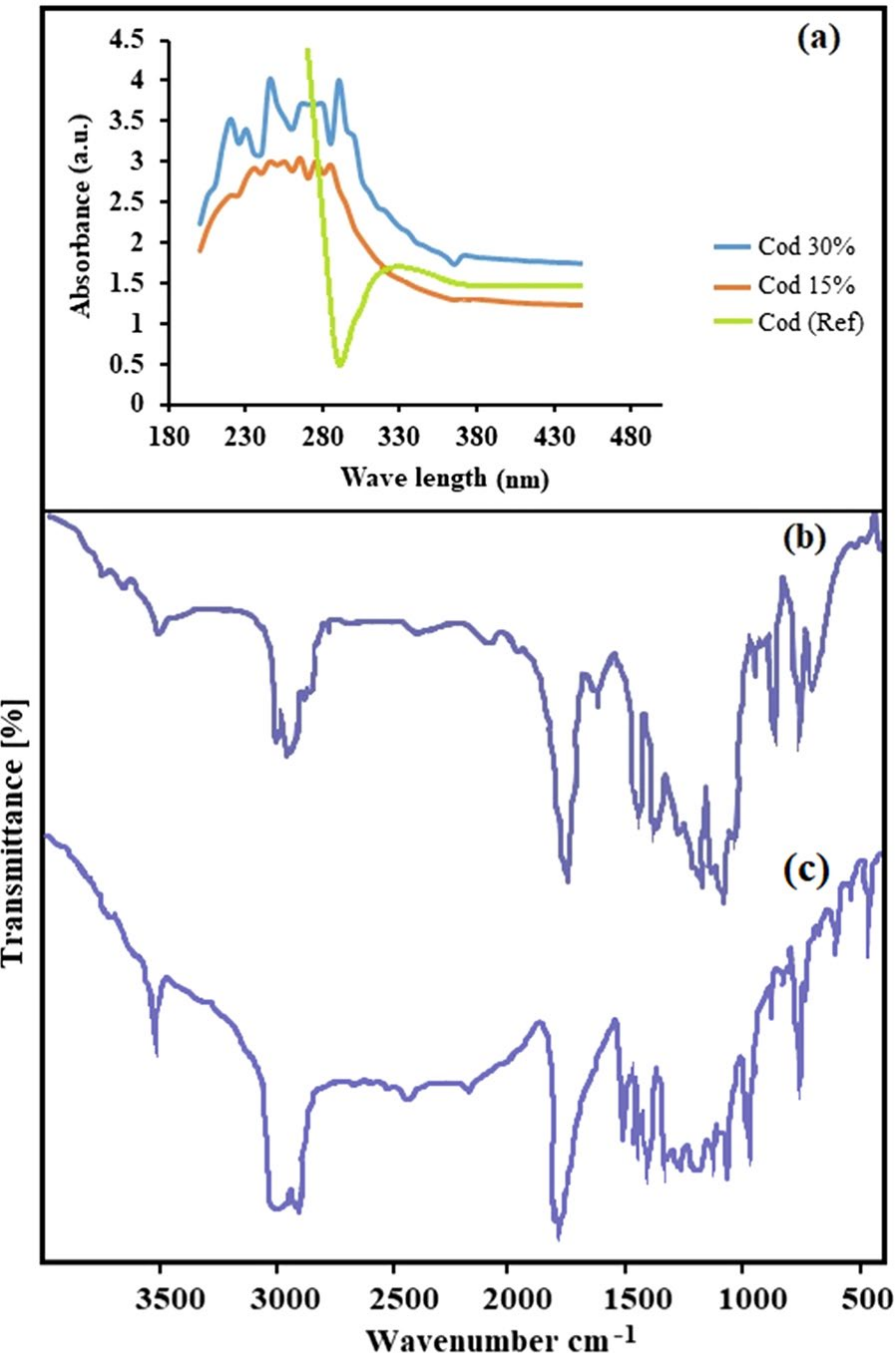
UV–Vis absorption spectra of poly lactic acid/chitosan nanofibers containing cod liver oil 30% w/w, 15% w/w and cod liver oil [62] (**a**) and FT-IR spectrum of the poly lactic acid/chitosan nanofibers containing 30% w/w (**b**) and 15% w/w (**c**) cod liver oil

UV–Vis as a general qualitative technique can be used to identify and confirm functional groups in a compound by matching the absorbance spectrum. Absorption in UV–Vis spectroscopy follows the Beer’s Law:

1 $${\text{A}} = \upvarepsilon \times {\text{b}} \times {\text{C}}.$$

where ε is the molar attenuation coefficient, b is path length, and C is concentration. UV–Vis absorption spectra of poly lactic acid/chitosan nanoscaffolds containing cod liver oil showed that, with increasing concentration from 15 to 30% w/w, absorbent peaks became noticeably more intense. However, the cod liver oil was not absorbed alone and it can be concluded that more loading of cod liver oil occurred in poly lactic acid/chitosan nanoscaffolds. Figure 3a demonstrates UV–vis absorption spectra of poly lactic acid/chitosan nanofibers containing cod liver oil of 30% w/w and 15% w/w compared with the cod liver oil [62]. Fourier transform infrared spectroscopy (FT-IR) is an analytical technique used to identify functional groups in materials. Figure 3b, c shows FT-IR spectrum of the prepared poly lactic acid/chitosan nanoscaffolds containing 30% w/w and 15% w/w cod liver oil in the region 400–4000 cm^− 1^, respectively. The absorption peaks at 3454 cm^− 1^ and 1630 cm^− 1^ regions could be attributed to the stretching and bending vibrations of O–H groups from chitosan and omega3 structures in the cod liver oil. The obtained peaks at 2884 cm^− 1^, 1650 cm^− 1^, and 1600 cm^− 1^ expressed the existence of stretching mode C-H, CH_2_ groups, C=O, C=C, and C–N regions in chitosan omega3 in cod liver oil and poly lactic acid structures. The reflectance at 3093 cm^− 1^ showed N–H band in chitosan. In general, the cod liver oil structures with forming chemical bonds are located in nanobio-polymeric nanoscaffold structures.

### In vivo study

Diabetic rats were evaluated in two ways; (a) blood glucose measurement by glucometer, rats with blood glucose above 200 mg/dl were considered diabetic and selected for further study, (b) the selected rats were examined for the appearance of diabetic symptoms including polyuria, overeating, and thirst and were assured of their diabetic status. As shown in the results and tables, in the group using nanofibers mixed with cod liver oil, the wound healing process (assessed by measuring the wound area) was investigated. It showed significantly better results than the group that used nanofiber alone or cod liver oil alone. It also appears that 30% cod liver oil supplementation with polyelactic acid/chitosan nanoscaffolds resulted in 94.5% wound healing on day 14, whereas 15% cod liver oil can heal wounds by about 86% on day 14, as presented in Table 2. Macroscopic changes of the wound were evaluated for treatment progress on days 0, 3, 7, and 14 after treatment and recorded by a photograph. The percentage of wound healing was calculated using the following formula:

2 $$Percentage\;of\;recovery = {{\left( {Surface\;wound\;on\;the\;first\;day - Surface\;wound\;in\;day\;X} \right)} \mathord{\left/ {\vphantom {{\left( {Surface\;wound\;on\;the\;first\;day - Surface\;wound\;in\;day\;X} \right)} {\left( {Surface\;wound\;on\;the\;first\;day} \right)}}} \right. \kern-\nulldelimiterspace} {\left( {Surface\;wound\;on\;the\;first\;day} \right)}} \times 100.$$

**Table 2:** Evaluation of wound healing rate in study groups studied on days 7 and 14

Percentage of recovery per day	Seven	Fourteenth
Control group	23.5	42.0
Nanofiber + cod liver oil 30%	64.8	94.5
Nanofiber + cod liver oil 15%	53.5	86.0
Poly lactic acid/chitosan nanofibers	34.1	60.0
Drug soluble group	39.5	59.9

**Fig. 4:**
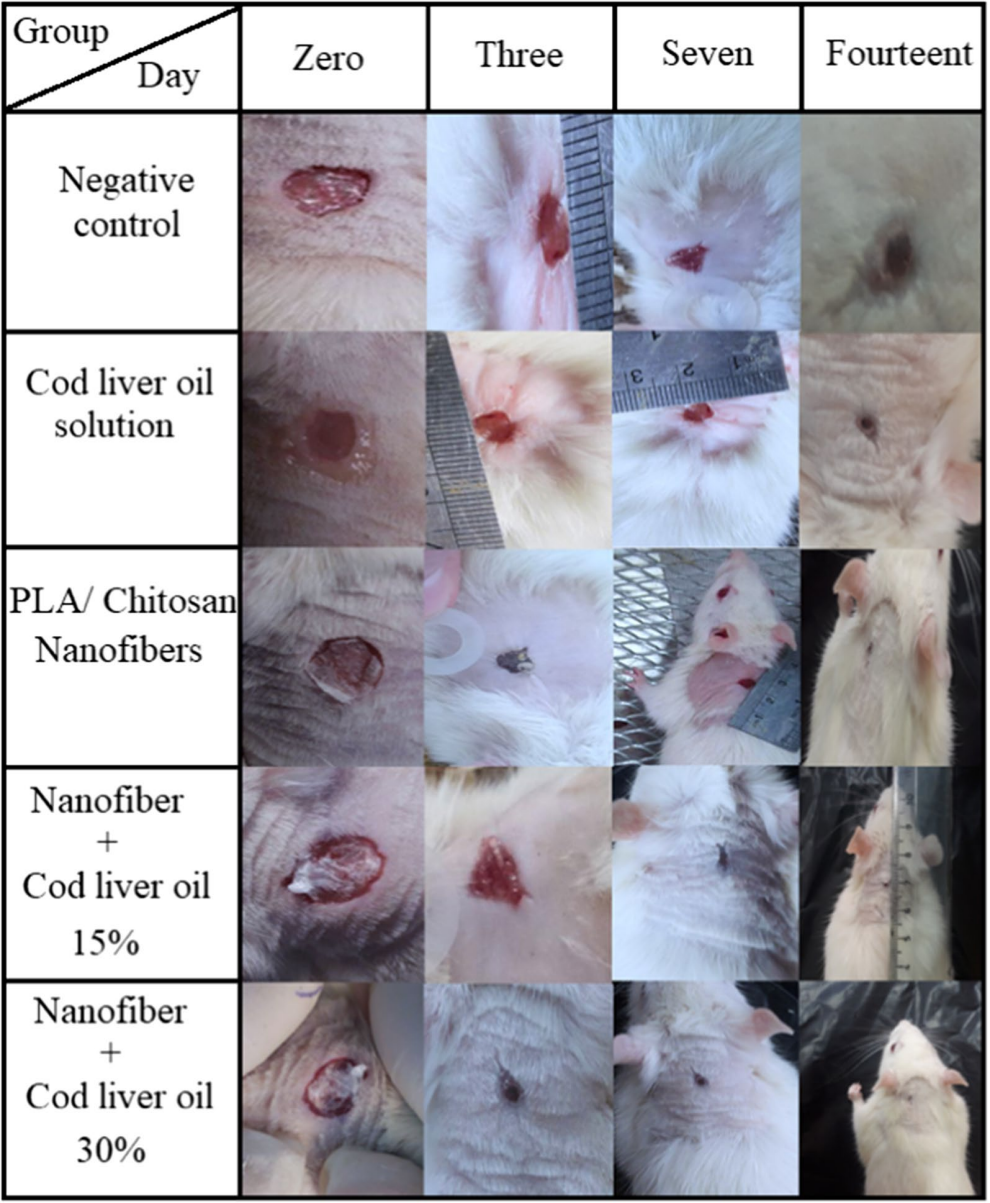
Wound area in five groups of animals studied on days 0, 3, 7 and 14 (mean ± sd; N = 3)

Figure 4 demonstrates a 14-day course examination of the wound surface and a photograph of the wound healing process. On days 0, 3, 7, and 14, the rate of wound healing in the nanoscaffold saline-treated mice with 30% cod oil was significantly different from that of the untreated control group and also from the other groups. The poly lactic acid/chitosan nanoscaffolds as bio-compatible and bio-degradable polymers could interact with skin cells and accelerate the healing process. Due to the high porosity of the nanoscaffold coating and the hydrogel-like properties of the polymers used, the coating swelled after the absorption of moisture and created a very small gap between the coating and the wound surface. On day 14, these characteristics were well observed and the nanoscaffold porosity property permitted oxygen to pass through the wound while keeping its surface moist.

**Fig. 5:**
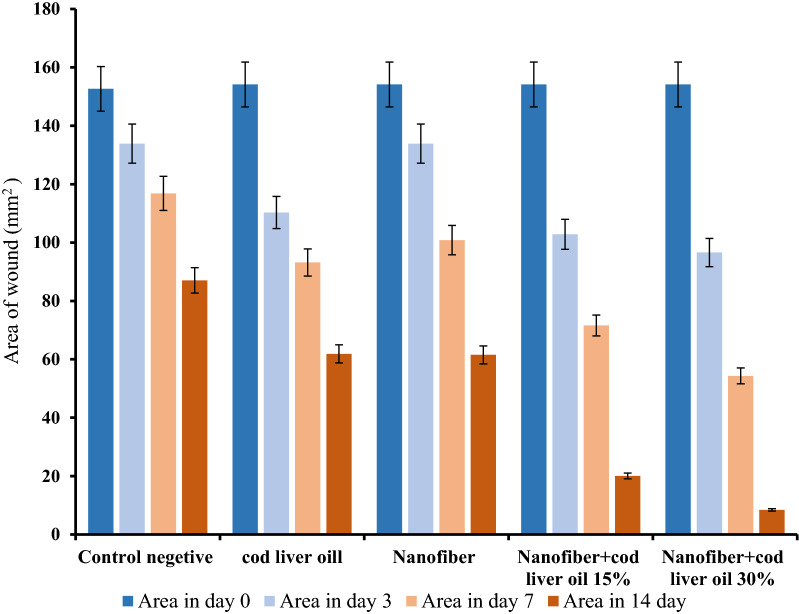
The wound healing process observed in the study groups

Wound area in five groups of animals studied on days 0, 3, 7, and 14 (mean ± sd; N = 3) is shown in Fig. 5. The area of the wound in the group with 30% cod oil was significantly less than the other groups; this indicated greater improvement in this group than in the other groups. The presence of bio-compatible nanofibers not only induced immune and allergic responses, but also made the body resemble the original tissue located in the wound. As a result, bio-chemical signals are needed to accelerate recovery and, eventually, wound healing will occur faster.

## Conclusion

In this research, the nanofibers scaffolding produced with electrospinning method was used to repair wounds on the skin of the rats. Macroscopic and microscopic studies were performed on the wounds to determine the efficacy of the produced nanoscaffolds after the desired time. The poly lactic acid/chitosan as bio-compatible and bio-degradable polymers scaffolding were designed for wound healing to be able to control drug (cod liver oil) release over a long period of time by improving the chemical structure. The poly lactic acid/chitosan nanoscaffolds containing 30% cod liver oil showed more healing and less wound area on day 14; this can be due to permeability and sufficient oxygen for tissue repair. Moisture retention of the wound medium for accelerating its healing, color change, and pH changes to keep the wound site safe from bacteria, contamination, and non-contamination was among the characteristics of the produced nanoscaffold.
